# An Array SPRi Biosensor for the Determination on PARP-1 in Blood Plasma

**DOI:** 10.3390/biomedicines11020602

**Published:** 2023-02-17

**Authors:** Zuzanna Zielinska, Lukasz Oldak, Joanna Kacperczyk-Bartnik, Ewa Koc-Żórawska, Marcin Żórawski, Piotr Laudanski, Ewa Gorodkiewicz

**Affiliations:** 1 Doctoral School of Exact and Natural Science, Faculty of Chemistry, University of Bialystok, 15-245 Bialystok, Poland; 2 Bioanalysis Laboratory, Faculty of Chemistry, University of Bialystok, 15-245 Bialystok, Poland; 3 2nd Department of Obstetrics and Gynecology, Medical University of Warsaw, 02-091 Warsaw, Poland; 4 Club 35, Polish Society of Gynecologists and Obstetricians, 53-125 Wrocław, Poland; 5 II Department of Nephrology and Hypertension with Dialysis Unit, Medical University of Bialystok, 15-276 Białystok, Poland; 6 OVIklinika Infertility Center, 01-377 Warsaw, Poland; 7 Women’s Health Research Institute, Calisia University, 62-800 Kalisz, Poland; 8 Department of Obstetrics, Gynaecology, Gynecology and Gynaecological Oncology, Medical University of Warsaw, 02-091 Warsaw, Poland

**Keywords:** SPRi biosensor, biomarkers, endometriosis, PARP-1

## Abstract

A biosensor was developed for the quantification of poly(ADP-ribose) polymerase-1 (PARP-1) in body fluids. An antibody specific for PARP-1 was placed on a chip with cysteamine (linker) and a gold layer. This biosensor has a linear response range (10–1000 pg∙mL^−1^) under appropriate pH conditions and with an antibody ligand concentration of 5 ng∙mL^−1^. Plasma samples were diluted with PBS buffer in appropriate quantities so that they fell within the linear range of the calibration curve. The biosensor exhibited suitable precision and accuracy, and good recovery (at levels from 95% to 105%). The method was validated by means of PARP-1 determinations in plasma samples from patients with endometriosis and a control group, using surface plasmon resonance imaging (SPRi) biosensors and an enzyme-linked immunosorbent assay (ELISA) test. The Spearman correlation coefficient was close to 1. PARP-1 may be a marker providing information about pathological changes in the body during endometriosis.

## 1. Introduction

**Figure 1:**
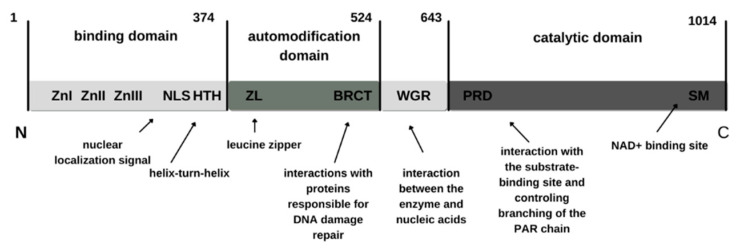
Structure of PARP-1. Sequence ends: the N-terminus and the C-terminus.

Poly(ADP-ribose) polymerase-1 (PARP-1) is an enzyme belonging to the family of eighteen poly(ADP-ribose) polymerases [1]. Its mass is 113 kDa, and it is found in the cell nucleus (often associated with chromatin), in cytoplasm [2], in Cajal bodies [3], and in blood plasma [4]. It participates in the proper formation of the nucleus, because a certain amount of PARP-1 is also present in the nuclear matrix. The gene that codes PARP-1 is located on the first chromosome of human cells, and is 23 exons long. It consists of automodification, binding, and catalytic domains. In its structure, we can distinguish the N-terminus and the C-terminus. The binding part of the enzyme is located at its N-terminal part. The automodification part contains a leucine zipper, which allows PARP-1 to interact with other proteins, and more specifically enables the heterodimerization and homodimerization of proteins [5]. There is also the BRCT domain, which supports the interaction of the enzyme with DNA, as well as with its protein partners such as poly(DP-ribose) polymerase-2 (PARP-2). The bonding domain has a mass of approximately 46 kDa and contains two zinc fingers, capable of activating PARP-1 in case of damage to the genetic material. ZnI participates in repair processes in case of damage to a single DNA strand, while ZnII is involved in the repair of both strands. These fingers are specialized in recognizing the structure of DNA [6,7]. The literature also mentions the presence of a third zinc finger, ZnIII, which is responsible for interactions between protein domains [8]. In the binding part, we can also distinguish the NLS (Nuclear Localization Signal) site, which enables the transport of freshly synthesized PARP-1 enzyme molecules to the cell nucleus. At a site called HTH (helix-turn-helix), PARP-1 binds to DNA. The catalytic domain of a protein presents a region of interaction between the enzyme and nucleic acids (WGR). It contains a catalytic center, which is an acceptor site for NAD+, where the first molecule of ADP-ribose is bonded. I Figure 1. Shows a structure of PARP-1.

**Figure 2:**
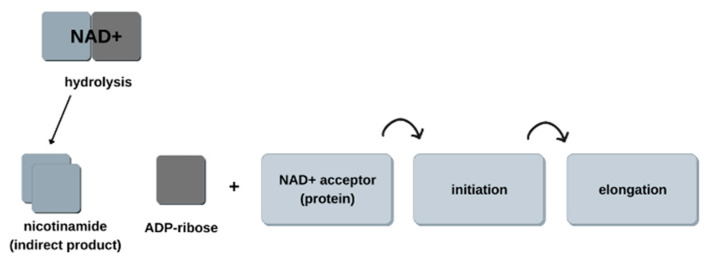
Diagram of the ADP-ribosylation process.

Many PARP enzymes, such as PARP-2, PARP-5, and PARP-10, are enzymatically active—they have a catalytic domain in their structure. They take part in processes related to ADP-ribosylation [5]. This is a post-translational protein modification process that affects many processes in the cell nucleus, such as transcription, DNA repair, and apoptosis [9]. PARP enzymes affect signaling pathways in the cell (PARP-10, PARP-14), transcription processes (PARP-3, PARP-7), as well as membrane organelles (PARP-8, PARP-16). During ADP-ribosylation, NAD+ molecules are transferred to target proteins [10]. NAD+ hydrolyzes and breaks down into ADP-ribose, with nicotinamide as a side product. Then, ADP-ribose is attached to the acceptor protein in linear, and in the next step in branched forms, with lengths of 200 to 400 units. This changes the properties of the modified proteins [5,6]. PARP-1 is directly involved in the catalysis of NAD+ decay to nicotinamide and ADP-ribose, and indirectly in DNA repair, primarily in detecting and signaling the occurrence of single and double DNA breaks. Its activity in the cell then increases up to 500 times, and binding with damaged DNA is a very fast process [8]. As a result of genotoxic or oxidative stress, the amount of poly(ADP-ribose) polymers in the cell nucleus increases, and they begin to reach the cytoplasm. These processes can proceed in two ways. The first is single-strand actions—cutting out incorrectly paired or single nitrogenous bases, as well as nucleotides. The second is repair within both strands. In this case, the genetic material is exchanged according to sequence homology [9]. Diagram of the ADP-ribosylation process is shown in Figure 2. 

**Figure A1:**
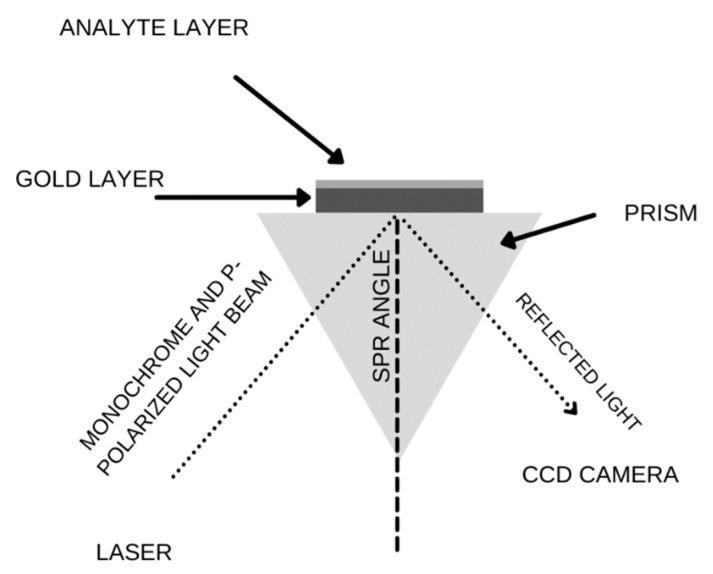
Diagram of the Kretschmann configuration.

Diagnostics very often focus on PARP-1’s operation. Through signaling mechanisms, it protects cancerous cells from damage, which in turn results in the disease’s development. Other mechanisms occur in the case of neurodegenerative diseases, or when regulating the action of other enzymes. Increased PARP-1 levels have been observed in the presence of many diseases. It plays a role in neurodegenerative diseases such as Alzheimer’s and Parkinson’s [11] due to competition with sirtuins (SIRT). SIRT-1, involved in the nervous system, is inactivated by PARP-1, which is the basis of the pathogenesis of nervous system diseases and aging [12,13]. PARP-1, through the regulation of inflammatory processes in the body, is more active in inflammatory diseases, including asthma, sepsis, arthritis, and atherosclerosis [14]. It is also possible to link the high activity of PARP-1 with the development of endometriosis, given the search for all possible immunological factors that may play a role in the pathogenesis of this disease [15,16]. However, neoplasms are currently in the spotlight, as they participate in the carcinogenesis and progression of neoplastic tumors [17]. In setting diagnostic goals, scientists have found a number of PARP-1 inhibitors, that can be used in many anti-cancer therapies, and are already applied in the treatment of gynecological cancers, such as endometrial, breast and ovarian cancer [18,19,20]. Therapies that are used to treat cancer deliver chemicals to cells that cause DNA damage. This damage is caught and repaired by repair mechanisms. PARP-1 activity then increases and the cell avoids apoptosis. It leads to the ineffectiveness of a given therapy. In the case of using a PARP-1 inhibitor, the repair processes will be inhibited, which in turn turns off the pathways providing information about cell damage. A rapid decrease in the enzyme’s activity leads to apoptosis. Such therapy is much more effective in destroying cancer cells [9]. Several methods for determining PARP-1 levels in biological material have been developed, including the quartz crystal microbalance (QCM), which showed PARP-1 levels to be low in normal ovarian cells (6.78 ng∙mL^−1^) and elevated in the presence of ovarian cancer (84.75 ng∙mL^−1^) [21]. Sensitivity to mass changes is one of the advantages of using a quartz microbalance for determinations, but it is not sensitive enough to determine PARP-1. H. Yang’s study used cetyltrimethylammonium bromide (CTAB)-coated gold nanorods (GNRs), which were positively charged, and interacted with negatively charged PARP-1. The quartz microbalance is a simple and easy-to-use apparatus, and can be miniaturized [21]. The ELISA test has also been used [16,20], as have differential pulse voltammetry (DPV) assays [4]. The ELISA assay shows higher PARP-1 concentrations in samples from endometriosis patients (0.45 ng∙mL^−1^) than in a control group (0.29 ng∙mL^−1^) [16]. However, it is a semi-quantitative method and requires the use of labeled antibodies. This is associated with higher costs due to the lack of diversity in the labeled antibodies that would specifically bind the antigen. Differential pulse voltammetry for the determination of PARP-1 has a long duration, and requires electrode modification and multi-stage testing [4,16]. In the present work, the image version of surface plasmon resonance (SPRi) was used. This method enables the quantitative determination of a substance without requiring the use of labels [22,23,24,25]. Compared with other methods, it enables measurements at picogram levels. It is characterized by a fast measurement, simple apparatus, and easy data analysis. The reagents used for testing are readily available [26]. The SPR technique is based on the resonance of plasmons, i.e., surface electrons of a metal or dielectric. It uses the Kretschmann configuration—a very thin layer of metal is illuminated, placed on a glass prism [27]. A diagram of the Kretschmann configuration is shown in Appendix A (Figure A1). This system is easy to build compared to other SPR configurations and does not require light to pass through the adsorbate. The excitation of plasmons with red or near-infrared light, in the wavelength range from 630 to 1200 nm, requires the use of a prism due to its suppression of total internal reflection [27].When a beam of p-polarized and monochromatic light strikes the metal (gold) layer, it causes the plasmons to resonate. The beam also has a certain wavelength and falls at a certain angle. When subsequent layers are applied to the metal, the angle that causes the SPR effect changes [28,29]. This allows the detection of adsorption and desorption processes on the surface, and also the determination of the amount of a given substance [27]. Due to the fact that the analyte of interest, which is contained in the sample, binds to a properly prepared matrix and is blocked on the metal surface, it is not necessary to clean and isolate the samples before the measurement. Since its first use, the surface plasmon resonance method has been enriched with further variants, and attempts have been made to combine it with other detection methods. SPR and SPRi differ in their detectors—in the case of the conventional SPR method, a regular photodetector is used and sensograms are plotted, while the SPRi method uses a CCD camera, which allows images to be obtained. In addition to the SPRi biosensors, in the literature other optical biosensors can be found that are used in drug development, diagnostics, or proteomics. They can be used to determine the affinity and kinetics of the formation of protein–ligand complexes. This is a label-free method based on the excitation of photonic crystal surface modes (PC SM) of a dielectric multilayer. This technique can screen the sample to capture hundreds of antibodies, due to the fact that they can be applied in 96 or 384 spots. Like SPRi biosensors, they operate with a certain accuracy and precision. This allows the determination of many compounds contained in the sample at the same time [30]. The aim of this study was to construct an SPRi biosensor that would be sensitive to PARP-1 and specific for that enzyme, for the purpose of its quantitative determination in blood plasma. In the constructed sensor, a specific PARP-1 antibody was immobilized on the gold layer of the chip, with cysteamine as a linker. Previously, the antibody had to be suitably activated using N-ethyl-N’-(3-dimethylaminopropyl)carbodiimide hydrochloride (EDC) and N-hydroxysuccinimide (NHS). Validation was carried out by measuring plasma samples from patients with endometriosis and from a control group. PARP-1 has not previously been determined by this method.

## 2. Experimental

### 2.1. Material and Reagents

The PARP-1 protein and a mouse monoclonal antibody specific for PARP-1 (ABcam, Cambridge, UK), EDC (N-ethyl-N’-(3-dimethylaminopropyl)carbodiimide hydrochloride) (SIGMA, Steinheim, Germany), N-hydroxysuccinimide (NHS) (Aldrich, Munich, Germany), carbonate buffer, cysteamine hydrochloride (2-aminoethanethiol) (Aldrich, Munich, Germany), buffered saline solution (PBS buffer) (Biomed, Lublin, Poland) and absolute ethyl alcohol (POCh, Gliwice, Poland) were used. The base of the biosensor is a plate with a gold layer (Ssens, Enschede, Netherlands). Human PARP ELISA kits (SunRedBio, Shanghai, China) were used, with a sensitivity of 7.282 ng∙L^−1^ and assay range of 8–2000 ng∙L^−1^.

### 2.2. Apparatus

**Figure A2:**
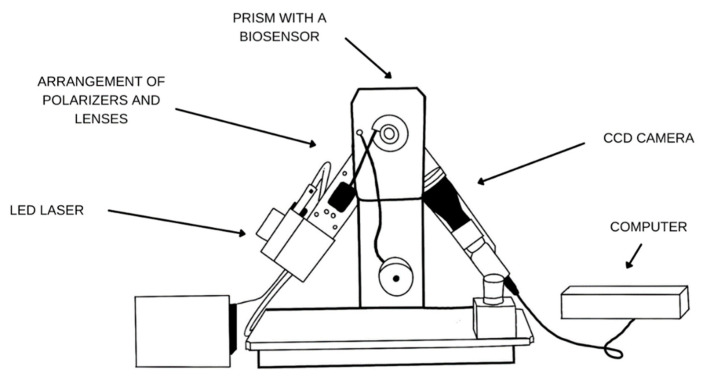
Diagram of the SPRi apparatus with its components.

The apparatus for measurements of the surface plasmon resonance imaging available in the Bioanalysis Laboratory, was used for the tests. The device consists of an LED laser emitting light of wavelength λ = 635 nm, polarizers and lenses, and a prism with a biosensor. The light reflected from the biosensor surface is collected by a 1.4 MP CCD camera and converted into an image. The construction elements are movable, and their angular range is from 30° to 75°. The optimal value of the SPR angle is selected individually for each biosensor during the ligand immobilization process. A diagram of the apparatus is given in Appendix A (Figure A2).

A scanning electron microscope (DualBeam Scios 2, Thermo Scientific, Waltham, MA, USA) was used, with an accelerating voltage of 2 kV and magnification of 50,000×.

### 2.3. Biological Material

The biological material was blood plasma collected in 2018–2019 from patients with endometriosis. The selected samples came from biobank created within project number: 6/6/4/1/NPZ/2017/1210/13522, financed by the Polish Ministry of Health. The research was approved by the bioethics committee (no. AKBE/132/2020).

### 2.4. Procedures

#### 2.4.1. Chip Preparation and Ligand Immobilization 

**Figure 3:**
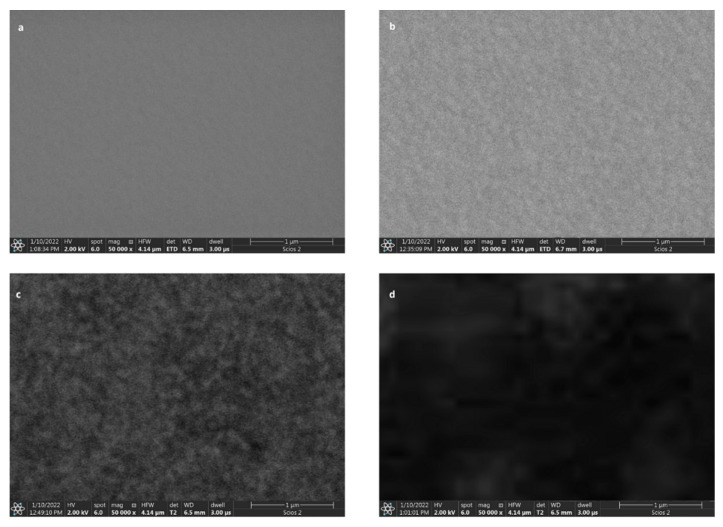
Scanning electron microscope images of the biosensor showing the process of monolayer formation on its surface: (**a**)—gold, (**b**)—cysteamine, (**c**)—antibody, (**d**)—PARP-1 protein (HV = 2.00 kV, mag = 50,000×, HFW = 4.14 µm).

**Figure A3:**
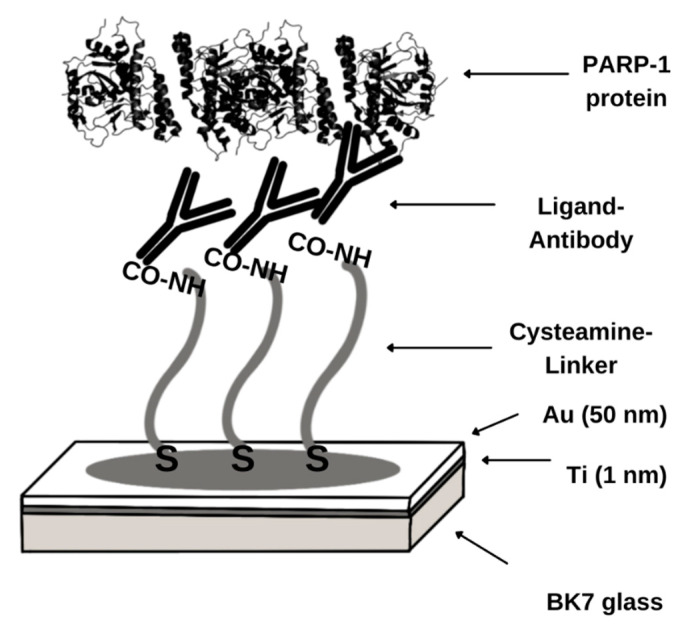
Diagram of the biosensor structure.

The proper operation of the sensor requires the immobilization of appropriate biological layers on the surface of the gold chip. The preparation of the biosensor base has been described in previous articles [29,31]. A diagram of the process of immobilization of layers on the chip surface is given in Appendix A (Figure A3). The base of the biosensor is made of BK7 glass, which is suitably polished and cleaned, and dried under a stream of argon. A layer of metals—titanium (1 nm) and gold (50 nm)—is sputtered onto this layer. A layer of photopolymer is then applied to isolate the twelve active sites (exposed gold layer) in each of the nine sample application sites. A hydrophobic mask is applied to prevent mixing of samples. A glass plate with a sputtered gold layer was immersed in a 20 mM cysteamine alcoholic solution for approximately 12 h. After this time the chip was rinsed with absolute ethanol and distilled water. Subsequently, the antibody-ligand (3.25 µL) was activated by the addition of EDC (15.6 µL), NHS (15.6 µL), and a carbonate buffer (6.25 µL), which allowed the antibody to bind to the cysteamine. The chip was incubated for one hour at 37 °C, and then washed with distilled water and HBS-ES buffer. After drying, 3 µL of sample (containing PARP-1) was applied for 10 min, and the biosensor was rinsed again. SEM (scanning electron microscope) photographs were taken to confirm the layer formation (Figure 3). The SEM images show how the surface of the chip changes on application of the layers that make up the biosensor.

#### 2.4.2. SPRi Measurement

**Figure A4:**
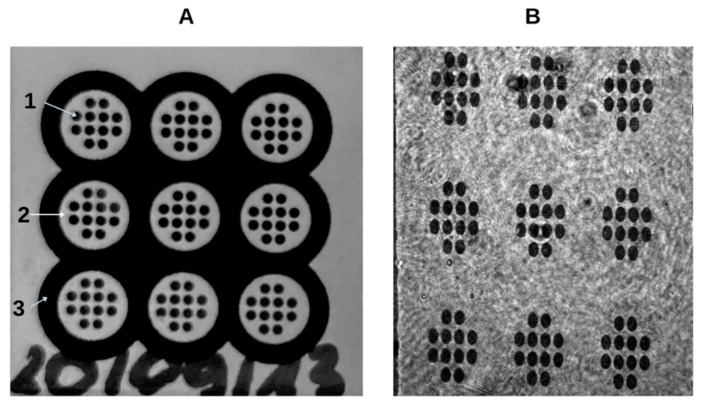
(**A**)–diagram of the chip (1–photopolymer, 2–gold surface, 3–hydrophobic paint); (**B**)–SPR image of the chip obtained using a CCD camera.

The SPRi signal is proportional to the amount of substance on the sensor’s surface over some analytically useful concentration range. After incubating the biosensor with the antibody, it was washed with distilled water and HBS-ES buffer. The chip prepared in this way was placed on the prism with the use of immersion oil. The next step was to determine the angle at which the SPR signal had the greatest intensity, that is, where the signal difference between the receptor and the analyte was the largest. The chip with the antibody (ligand) layer was measured. The samples (3 µL) containing PARP-1 were applied and left for 10 min. The surface was washed several times with HBS-ES buffer and distilled water. In each of the nine places for sample application, there are twelve active sites, i.e., exposed parts of the gold, enabling twelve repetitions of sample measurement. Successive measurements were made and saved as images. A schematic diagram of the chip and an image of the SPR chip obtained with a CCD camera are shown in Appendix A (Figure A4). Quantification is derived from the integrated contrast values of the images obtained from the CCD camera. Then, at a specific point of the biosensor’s active site, the evaluation of two-dimensional SPRi images and the conversion of numerical signals into a quantitative signal were performed. The ImageJ software (version 1.53, National Institutes of Health, NIH) was used for these mathematical operations. After the measurement procedure, the chips are suitably washed with NaOH (2 M) and triton X-100 solution at 60 °C. They are then rinsed in distilled water heated to 60 °C and dried in a stream of argon. This enables alkaline hydrolysis of the peptide bond between the antibody and the cysteamine immobilized on the surface of the chip. One gold chip can therefore be used again, at least six times.

#### 2.4.3. ELISA Measurement

The determination of the PARP-1 concentration in blood plasma was performed using a human PARP-1 enzyme-linked immunoassay from an ELISA kit, according to the manufacturer’s instructions.

#### 2.4.4. Preparation of Biological Samples

Blood plasma from patients with endometriosis were diluted 10 times, and control samples 2 times, with 0.15 M PBS buffer. This was done to bring the concentrations within the linear range of the calibration curve.

## 3. Results and Discussion

### 3.1. Optimization of the Concentration of the Ligand (Antibody) Monolayer

**Figure A5:**
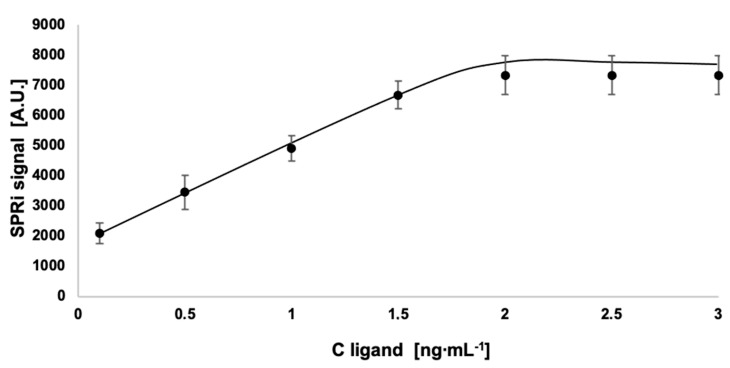
Surface saturation curves. Concentration of PARP-1 protein = 5 ng∙mL^−1^, pH = 7.4.

The determination of the appropriate ligand-antibody concentration was performed at a constant concentration of PARP-1 (5 ng∙mL^−1^) and pH = 7.4. The antibody concentrations ranged from 0.1 to 5 ng∙mL^−1^. The preparation and activation of the murine monoclonal antibody are described in Section 2.4.1. The results are given in Appendix A (Figure A5).

The SPRi signal and antibody concentration increase until the biosensor surface is completely saturated. Above 2 ng∙mL^−1^, ligand binding no longer takes place on the biosensor surface. Therefore, 2 ng/mL was chosen as the optimal concentration of the antibody used for the assay.

### 3.2. Influence of pH on the Activity of PARP-1

**Figure A6:**
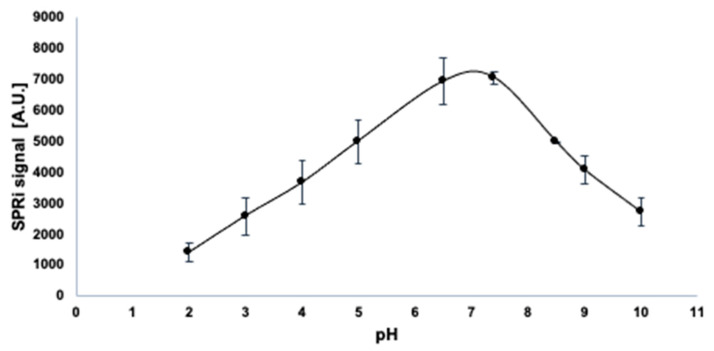
Influence of pH solution on SPRi signal. Concentration of PARP-1 = 5 ng∙mL^−1^, antibody concentration = 2 ng∙mL^−1^.

Tests were conducted at pH values ranging from 2 to 10, using a constant concentration of PARP-1 (5 ng∙mL^−1^) and a constant concentration of ligand (2 ng∙mL^−1^). The results, in the form of a curve, are given in Appendix A (Figure A6). The sensor exhibits a maximum SPRi signal at a pH of 6.5 to 7.5.

### 3.3. Analytical Response to PARP-1 Concentration

**Figure 4:**
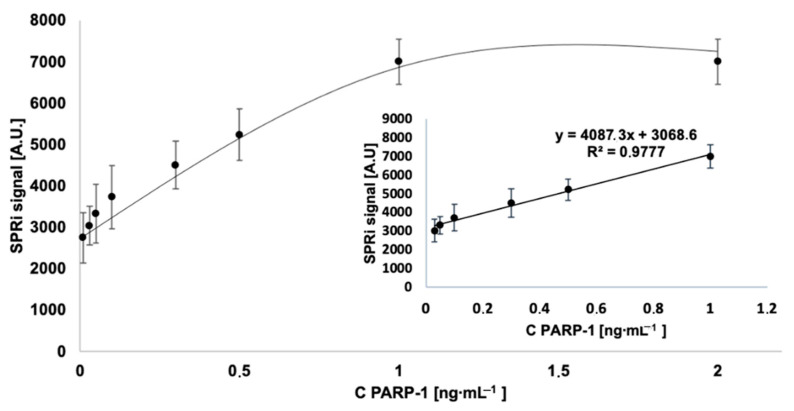
Calibration curves. Concentration of antibody = 2 ng∙mL^−1^, pH = 7.4.

A calibration curve was used to test the analytical response of the biosensor. The SPRi signal was measured over a PARP-1 concentration range from 0.01 to 2 ng∙mL^−1^. The procedures of the experiment are described in Section 2.4.2. The results are shown in Figure 4. The full range of the calibration curve contains a linear fragment that is analytically useful. The limit of detection (LOD) is 7 pg∙mL^−1^ (1). This is the lowest point on the calibration curve, excluding the blank. The limit of quantification (LOQ) is 20 pg∙mL^−1^ (2).

(1) $$\mathrm{LOD}=\frac{3.3*SD}{a}$$

(2) $$\mathrm{LOQ}=\frac{10*SD}{a}$$

LOD—limit of detection
LOQ—limit of quantification
*SD*—standard deviation of the blank
*a*—slope of the calibration curve

### 3.4. Precision and Accuracy of the Determination of PARP-1 Concentration 

Precision and accuracy are used to determine the suitability of an analytical procedure. Concentrations of PARP-1 with values of 10, 500, and 1000 pg∙mL^−1^ were used, corresponding to the lowest, middle, and highest concentrations in the linear range, respectively. The number of measurements was 24 for each concentration. The results are shown in Table 1. The recovery for three samples was found to be at a good level (from 95% to 105%); the results show the biosensor to have good precision and accuracy.

### 3.5. The Influence of Interferents on the Analytical Signal

Patients’ plasma and blood contain many components that may significantly affect the test results. It is important that the result corresponds only to the presence of the tested compound, in this case PARP-1. Naturally occurring albumin, as well as PARP-2, were selected as potentially interfering compounds. PARP-2 exhibits similar properties to PARP-1. Solutions of PARP-1 mixed with the selected interferents were then placed on the sensor. The results are shown in Table 2. The recovery values indicate a high analytical specificity; no negative effect of the matrix is observed.

### 3.6. PARP-1 Determination in Real Samples

**Figure 5:**
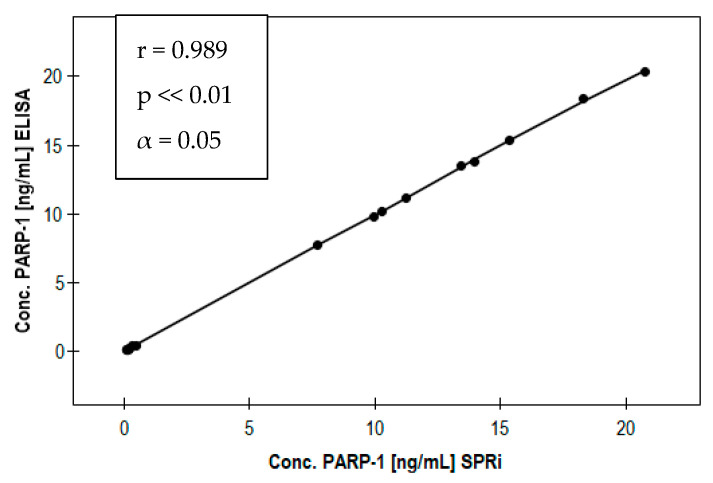
Correlation of the results for the patient and control groups obtained with the SPRi biosensor and ELISA.

The developed method was used for the determination of PARP-1 in nine plasma samples from patients with endometriosis, and in nine samples from patients in whom, apart from minor gynecological complaints, no endometriosis or cancer was detected (control group). The aim was to prove the usefulness of the method in practice. The assay procedure was as described in previous sections. The results are given in Appendix A (Table A1). Plasma samples were diluted with PBS buffer to bring them within the linear range of the calibration curve. The results were compared with those obtained using a standard method, the ELISA test. The results are shown in Appendix A (Table A1). The graph in Figure 5 shows the correlation curve of the results for the SPRi biosensor method and the ELISA test. The Spearman correlation coefficient was 0.989. The parameter *p* << 0.01 indicates the high statistical significance of the differences between the concentrations in the group of sick patients and in the control group.

## 4. Conclusions

A PARP-1-sensitive biosensor with SPRi detection was constructed for the determination of the enzyme in blood plasma samples. Images from a scanning electron microscope confirmed the formation of layers on the surface of the chip. The construction of a new biosensor characterized by a high precision of measurements and an appropriate sensitivity required a series of measurements to determine appropriate validation parameters. First, the optimal ligand concentration (2 ng∙mL^−1^) was established. It was also found that the highest SPR signal is obtained at pH from 6.5 to 7.5. The limit of detection (LOD) for PARP-1 was determined to be 0.007 ± 0.003 ng∙mL^−1^, and the limit of quantification (LOQ) to be 0.02 ± 0.01 ng∙mL^−1^. The equation of the calibration curve was determined for the linear response range. The usefulness of thes constructed biosensor for determinations in body fluids was tested by measuring plasma samples from patients in a control group and from patients suffering from endometriosis. Literature data show that poly(ADP-ribose)-1 polymerase enzymes are overexpressed in endometrial cancer, and also during endometriosis. Increased amounts of the enzyme have been reported mainly in advanced cases of endometriosis [32,33]. Other studies show that PARP-1 may play an active role in endometrial cancer, which may be secondary to endometriosis, by increasing oxidative stress. In the present study, the concentrations of PARP-1 in the control samples did not exceed 0.5 ng∙mL^−1^, while the concentration of the enzyme in samples from endometriosis patients was much higher, reaching as much as 20 ng∙mL^−1^. The parameter *p* < 0.01 indicates the high statistical significance of the differences between the concentrations observed in the patient group and in the control group. In a comparative analysis of the results obtained by the SPRi method and the ELISA test, the Spearman correlation coefficient was found to be close to 1. The results of the study show that PARP-1 may be a marker of endometriosis, exhibiting a higher concentration in that disease, but it can also provide information about neoplastic changes.

## Figures and Tables

**Table 1:** Precision and accuracy of methods for the determination of PARP-1 concentration (*n* = 24).

C_theoretical_ [ng∙mL^−1^]	C_found_ [ng∙mL^−1^]	SD [ng∙mL^−1^]	Recovery [%]	RSD [%]
0.010	0.099	0.0001	99	0.0101
0.500	0.5007	0.0127	100.14	0.0254
1.000	1.0335	0.0398	103.35	0.0385

**Table 2:** Selectivity of the investigated method of PARP-1 determination.

Potential Interferent	C_PARP-1_ vs. C_interferent_	C_found_ [ng∙mL^−1^]	Recovery [%]
Human albumin	1:1	0.517	103
	1:10	0.512	102
	1:100	0.500	100
PARP-2 protein	1:1	0.527	105
	1:10	0.507	101
	1:100	0.499	99

## Appendix A

**Table A1:** Comparison of results for blood plasma samples from patients with endometriosis (resarch group) and the control group, obtained by the SPRi method and ELISA as the reference method.

Patients Group		
Number of Sample	SPRi	ELISA
	Concentration of PARP-1 [ng∙mL^−1^]	Concentration of PARP-1 [ng∙mL^−1^]
1A	11.046	11.140
2A	18.024	18.350
3A	7.679	7.744
4A	13.011	13.490
5A	20.576	20.360
6A	15.294	15.390
7A	9.459	9.750
8A	13.875	13.780
9A	10.233	10.180
Control group		
Number of sample	SPRi	ELISA
	Concentration of PARP-1 [ng∙mL^−1^]	Concentration of PARP-1 [ng∙mL^−1^]
1	0.197	0.196
2	0.163	0.160
3	0.113	0.141
4	0.140	0.131
5	0.155	0.160
6	0.144	0.115
7	0.209	0.222
8	0.369	0.386
9	0.427	0.451
